# Allogeneic Human Mesenchymal Stem Cell Infusions for Aging Frailty

**DOI:** 10.1093/gerona/glx056

**Published:** 2017-04-21

**Authors:** Samuel Golpanian, Darcy L DiFede, Aisha Khan, Ivonne Hernandez Schulman, Ana Marie Landin, Bryon A Tompkins, Alan W Heldman, Roberto Miki, Bradley J Goldstein, Muzammil Mushtaq, Silvina Levis-Dusseau, John J Byrnes, Maureen Lowery, Makoto Natsumeda, Cindy Delgado, Russell Saltzman, Mayra Vidro-Casiano, Marietsy V Pujol, Moisaniel Da Fonseca, Anthony A Oliva, Geoff Green, Courtney Premer, Audrey Medina, Krystalenia Valasaki, Victoria Florea, Erica Anderson, Jill El-Khorazaty, Adam Mendizabal, Pascal J Goldschmidt-Clermont, Joshua M Hare

**Affiliations:** 1 The Interdisciplinary Stem Cell Institute and; 2 Department of Surgery, University of Miami Miller School of Medicine, Florida.; 3 Longeveron LLC, Miami, Florida.; 4 Department of Medicine, University of Miami Miller School of Medicine, Florida.; 5 The Emmes Corporation, Rockville, Maryland.

**Keywords:** Regenerative medicine, Cell-based therapy, Physical function, Inflammation

## Abstract

**Background:**

Impaired endogenous stem cell repair capacity is hypothesized to be a biologic basis of frailty. Therapies that restore regenerative capacity may therefore be beneficial. This Phase 1 study evaluated the safety and potential efficacy of intravenous, allogeneic, human mesenchymal stem cell (allo-hMSC)-based therapy in patients with aging frailty.

**Methods:**

In this nonrandomized, dose-escalation study, patients received a single intravenous infusion of allo-hMSCs: 20-million (*n* = 5), 100-million (*n* = 5), or 200-million cells (*n* = 5). The primary endpoint was incidence of any treatment-emergent serious adverse events measured at 1 month postinfusion. The secondary endpoints were functional efficacy domains and inflammatory biomarkers, measured at 3 and 6 months, respectively.

**Results:**

There were no treatment-emergent serious adverse events at 1-month postinfusion or significant donor-specific immune reactions during the first 6 months. There was one death at 258 days postinfusion in the 200-million group. In all treatment groups, 6-minute walk distance increased at 3 months (*p* = .02) and 6 months (*p* = .001) and TNF-α levels decreased at 6 months (*p* < .0001). Overall, the 100-million dose showed the best improvement in all parameters, with the exception of TNF-α, which showed an improvement in both the 100- and 200-million groups (*p* = .0001 and *p* = .0001, respectively). The 100-million cell-dose group also showed significant improvements in the physical component of the SF-36 quality of life assessment at all time points relative to baseline.

**Conclusions:**

Allo-hMSCs are safe and immunologically tolerated in aging frailty patients. Improvements in functional and immunologic status suggest that ongoing clinical development of cell-based therapy is warranted for frailty.

Frailty is a medical syndrome characterized by age-related diminished physiologic function, endurance, and strength that can be secondary to a multitude of factors and is associated with a high risk of hospitalizations, worse clinical outcomes, dependency, and mortality (1,2). The overall prevalence of frailty is estimated to be 9.9% and is more common in females as well as those with chronic disease (3). The etiology of frailty is multifaceted, with an age-related decline in either physical, psychological, or a combination of the two components at the helm of the disease process (1). As a result, patients with frailty will often experience a reduction in “life span” and a corresponding increase in dependence on others for activities of daily living. Over time, the elderly and frail individuals in particularly, begin to lack the ability to adapt to adverse environmental stressors secondary due to their lack of “physiologic reserve,” which leads to impaired function and increases their vulnerability to death (4–7). Frail patients can be classified based on severity, as frailty has been characterized as a phenotypic spectrum through which patients transition (8).

Therapeutic interventions for aging frailty have mainly focused on exercise, nutritional supplements, and multidisciplinary methods (9,10). The goal of any potential therapy for frailty is to extend the well-being and ability of a patient to regenerate functionality. In this regard, one of the key hypotheses of the aging frailty is an accelerated depletion of endogenous stem cells, thereby contributing to a reduced ability to regenerate or repair organs and tissues (11,12). Accordingly, a cell-based, regenerative treatment strategy could ameliorate signs and symptoms of frailty (13,14–16).

Bone marrow–derived human mesenchymal stem cells (hMSCs) home to sites of injury, reduce inflammation and fibrosis, stimulate endogenous stem cells, and contribute to tissue regeneration (17). Clinical studies have demonstrated that hMSCs improve cardiac structure and function in patients with acute myocardial infarction (18) and ischemic and nonischemic heart failure (19,20). Importantly, hMSCs are immunomodulatory (21) and data from multiple clinical trials show that allogeneic hMSCs are safe (17–20,22) irrespective of age (23). Increased chronic inflammation (24,25) and declines in cardiovascular reserve (26) have been identified in aging frail patients. These features of aging frailty support the hypothesis that hMSCs ameliorate or improve frailty.

The purpose of this Phase 1 pilot study, Allogenei*C* Human Mesenchymal Stem Cell in Patients with Aging F*R*ail*T*y via Intraveno*US* Delivery (CRATUS) (27), was to evaluate the safety and tolerability of allogeneic hMSCs (allo-hMSCs) in patients with aging frailty and to explore domains of treatment efficacy of allo-hMSCs through the reduction of signs and symptoms of aging frailty. The study also sought to gain insight regarding optimal dosing of allo-hMSCs and potential mechanistic properties of intravenous allo-hMSC therapy in frailty vis-à-vis immune monitoring and measurements of changes in systemic biomarkers.

## MATERIALS AND METHODS

The Allogenei*C* Human Mesenchymal Stem Cell in Patients with Aging F*R*ail*T*y via Intraveno*US* Delivery (CRATUS) Study (www.clinicaltrials.gov: #NCT02065245) was a nonrandomized, nonblinded, escalated dose pilot Phase 1 study in which a total of 15 patients were enrolled for an infusion of human donor bone marrow–derived allo-hMSCs, delivered via peripheral intravenous infusion, as previously described in detail (27). Patients were scheduled to receive 20-million (Group 1, *n* = 5), 100-million (Group 2, *n* = 5), or 200-million (Group 3, *n* = 5) allo-hMSCs.

The study was approved by the Internal Review Board (IRB) at the University of Miami Miller School of Medicine (IRB approval #: 20130646) and monitored by a Data and Safety Monitoring Board. All subjects provided written informed consent before participating. Table 1 lists the complete study inclusion and exclusion criteria. See Supplementary Material for full description of the Methods.

**Table 1.** Key Inclusion and Exclusion Criteria for Enrolled Subjects

Key Inclusion Criteria
• Provide written informed consent
• Subjects age ≥60 and ≤95 y
• Show signs of frailty apart from a concomitant condition as assessed by the Investigator with a frailty score of 4 to 7 using the Clinical Frailty Scale (29)
Key Exclusion Criteria
• Score of ≤24 Mini-Mental State Examination
• Inability to perform any of the assessments required for endpoint analysis
• Serious comorbid illness or any other condition that, in the opinion of the investigator, may compromise the safety or compliance of the subject or preclude successful completion of the study
• Have a nonpulmonary condition that limits life span < 1 y
• Have a clinical history of malignancy within 5 y (ie, subjects with prior malignancy must be disease free for 5 y), except curatively-treated basal cell carcinoma, squamous cell carcinoma, melanoma in situ or cervical carcinoma, if recurrence occurs.

## Results

Study subjects (*n* = 15) had a 2:1 male to female ratio and an average age of 78.4 ± 4.7 years (Table 2). Over half of the patients (*n* = 8) were considered “vulnerable” with a Clinical Frailty Score (28,29) of 4, whereas the remainder scored a 5 (“mild” frailty; *n* = 6) or 6 (“moderate” frailty; *n* = 1). At baseline, the mean 6-minute walk distance (6MWD) and forced expiratory volume in 1 second (FEV1) were 382.6 ± 102.7 m and 2.3 ± 0.4 L, respectively. There was no significant difference between patients of each cell-dose group in either of these parameters. The average Mini-Mental State Examination (MMSE) score in this study cohort was 28.5 ± 1.4. Blood laboratory counts between patients were similar at baseline.

**Table 2.** CRATUS Phase 1: Subject Baseline Characterization

Characteristics	20M MSCs (*N* = 5)	100M MSCs (*N* = 5)	200M MSCs (*N* = 5)	Total (*N* = 15)
Gender				
Male	2	5	3	10
Female	3	0	2	5
Race				
White	5	5	5	15
Ethnicity				
Not Hispanic or Latino	5	5	5	15
Age at infusion (years)	79.5 ± 5.2	76.2 ± 4.9	79.4 ± 4.0	78.4 ± 4.7
Clinical Frailty Score (29)				
4 (“Vulnerable”)	2	4	2	8
5 (“Mild”)	2	1	3	6
6 (“Moderate”)	1	0	0	1
Average	4.8	4.2	4.6	4.53
6MWD, mean ± *SD*	430.8 ± 77.0	331.8 ± 109.2	385.2 ± 113.7	382.6 ± 102.7
FEV1 (L), mean ± *SD*	2.4 ± 0.5	2.5 ± 0.3	2.1 ± 0.4	2.3 ± 0.4
MMSE, median (min, max)	29 (25, 30)	28 (28, 29)	29 (28, 30)	29 (25, 30)
TNF-α, mean ± *SD*	4.1 ± 2.2	5.3 ± 1.9	5.9 ± 2.2	5.1 ± 2.1
SF-36 MCS, mean ± *SD*	45.4 ± 8.8	41.5 ± 9.2	42.6 ± 7.7	43.1 ± 8.1
SF-36 PCS, mean ± *SD*	50.0 ± 8.6	40.9 ± 8.5	44.5 ± 5.8	45.1 ± 8.1
EQ-5D, median (min, max)	6 (5, 7)	8 (7, 10)	8 (1, 9)	7 (1, 10)

*Note:* FEV1 = Forced expiratory volume in 1 s; M = million; MCS = Mental Component Score; MMSE = Mini-Mental State Examination; 6MWD = 6-Minute Walk Distance; PCS = Physical Component Score; SF-36 = 36-Item Short Form Health Survey; TNF = tumor necrosis factor.

Allo-hMSCs were well tolerated in all three cell-dose groups with no treatment-related serious adverse events (SAE) observed during the 12-month postinfusion follow-up period. There were no persistent cardiopulmonary signs or symptoms observed immediately following infusion. Supplementary Table 1 and Table 3 list the adverse events (AE) and SAE’s for this trial. Two subjects in the 20-million cell-dose group were hospitalized on Day 102 and 311 postinfusion for an SAE (uncontrolled hypertension and hypercalcemia, respectively); however, both events were considered unrelated to the product. Additionally, one infusion-related AE (intravenous infiltration) occurred in the 20-million cell-dose group, which was considered mild in severity and soon thereafter resolved. One subject in the 200-million cell-dose group experienced a sudden cardiac death 258 days postinfusion, which was determined to be unrelated to the study product.

**Table 3.** CRATUS Phase 1: Overall AE/SAE Summary

	Allo-20M (*N* = 5)	Allo-100M (*N* = 5)	Allo-200M (*N* = 5)
1 mo postinfusion			
AEs—*n* (%) (number of events)	2 (40.0%) (3)	1 (20.0%) (1)	2 (40.0%) (2)
SAEs—*n* (%) (number of events)	0	0	0
6 mo postinfusion			
AEs—*n* (%) (number of events)	5 (100.0%) (11)	3 (60.0%) (4)	3 (60.0%) (5)
SAEs—*n* (%) (number of events)	1 (20.0%) (1)	0	0
12 mo postinfusion			
AEs—*n* (%) (number of events)	5 (100.0%) (14)	4 (80.0%) (6)	3 (60.0%) (6)
SAEs—*n* (%) (number of events)	2 (40.0%) (2)	1 (20.0%) (1)	1 (20.0%) (1)^a^

*Note:* AE = adverse events; M = million; SAE = serious adverse events.

^a^This participant experienced a sudden cardiac death 258 d postinfusion, determined to be unrelated to study product.

**Figure 1.**
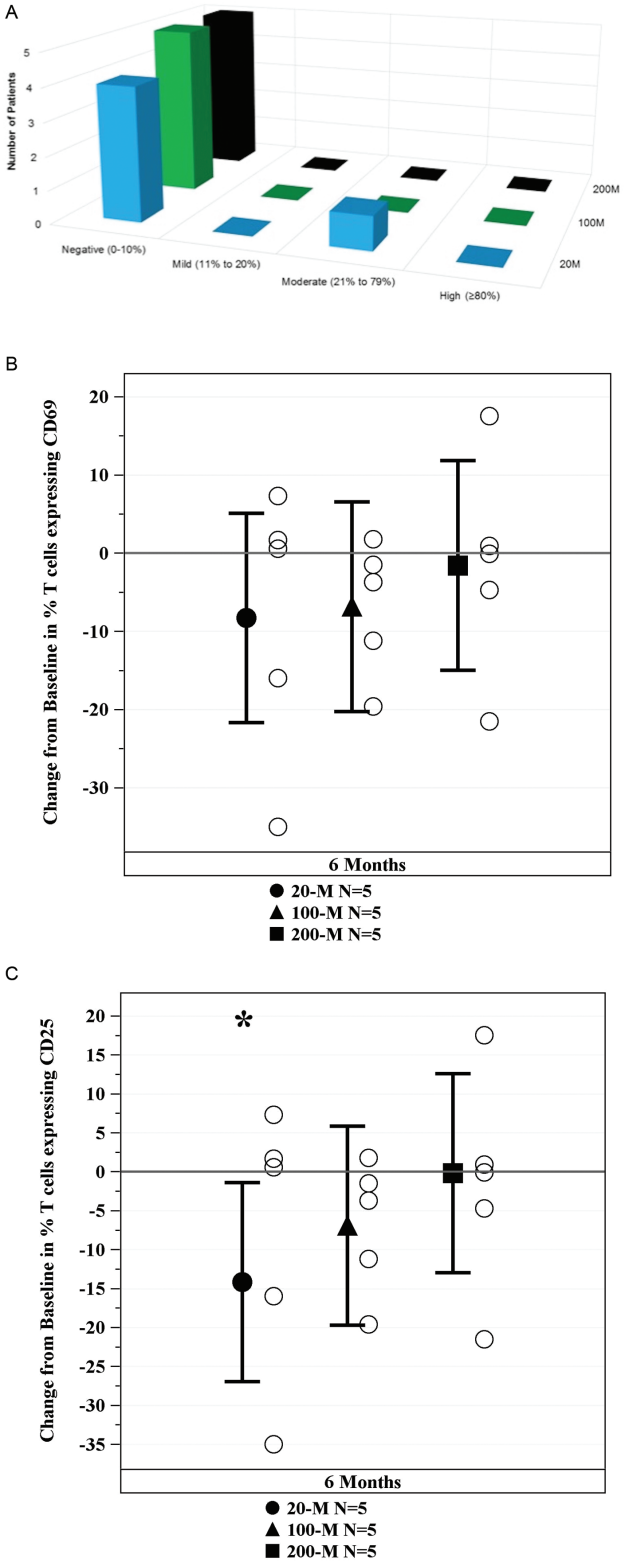
Immune monitoring. (A) Effects of mesenchymal stem cells (MSCs) on cPRA. Change in Calculated Panel Reactive Antibody (cPRA) assay from day 1 to 6 months demonstrates that only one subject in the 20-million (M) arm had a moderate donor-specific cPRA reaction and no subjects in the 100M and the 200M group had any reaction. (B) Effects of MSCs on early T cell activation. Change in T cells expression of early activation marker CD69 demonstrates that allo-hMSCs in all treatment arms suppresses early T cell activation. (C) Effects of MSCs of late/chronic T cell activation. Change in T cells expression of late/chronic activation marker CD25 demonstrates that allo-hMSCs in all treatment arms did not induce late/chronic T cell activation and the 20 million dose significantly suppressed activation (*p = .03) at 6 mo as compared to baseline.

Immunologic response to allo-hMSCs was assessed using panel reactive antibodies (PRA) testing. The calculated PRA (cPRA) was assessed at Day 1 and 6 months postinfusion. The change in percent cPRA depicts that only one patient in the 20-million allo-hMSC group developed a new donor-specific moderate (26%) cPRA reaction (Figure 1A). There were no signs of early T cell activation (CD69) at 6 months post-allo-hMSC as compared to baseline in all treatment groups (20-million: −8.28%, 95% confidence interval [CI] −21.68–5.12, *p* = .20; 100-million: −6.84%, 95% CI −20.24–6.56, *p* = .29; 200-million: −1.56%, 95% CI −14.97–11.84, *p* = .80; Figure 1B). Lastly, allo-hMSCs did not induce late/chronic (CD25) T cell activation in any of the treatment groups. Of note, the late/chronic T cell activation was significantly reduced in the 20-million group (20-million: −14.18%, 95% CI −26.96–−1.40, *p* = .03), although not in the other treatment groups at 6 months post-allo-hMSC as compared to baseline (100-million: −6.9%, 95% CI −19.68–5.88, *p* = .26; 200-million: −0.18, 95% CI −12.96–12.60, *p* = .98; Figure 1C). In summary, all 15 subjects tolerated the infusions well, with only one subject (20-million group) developing mild to moderate donor-specific antibodies.

**Figure 2.**
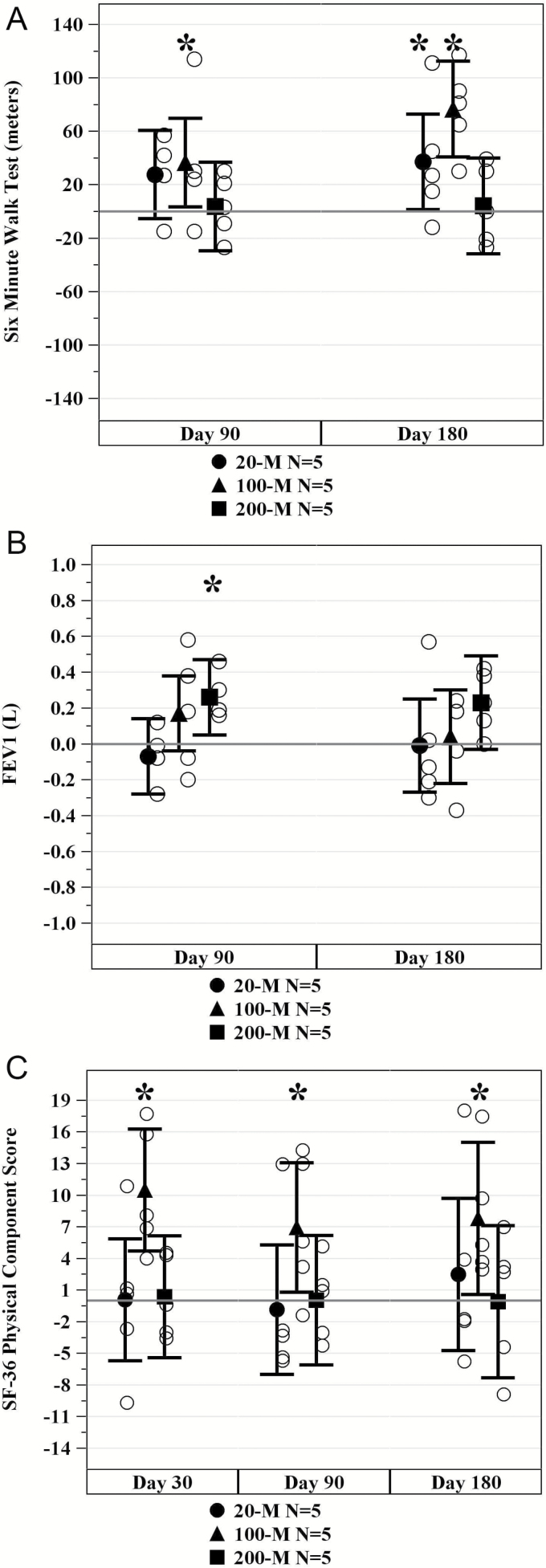
Effects of mesenchymal stem cells (MSCs) on physical markers of frailty. (A) Change in 6-minute walk distance (6MWD). The average 6MWD significantly improved from baseline for subjects in the 20-million (M) (at 6 mo, *p* = .043) and 100M arms (at 3 mo, *p* = .033 and at 6 mo, *p* = .0005) but not the 200M arm. *p Values are for within group versus baseline. (B) Change in forced expiratory volume in 1 s (FEV1). The average FEV1 significantly improved for subjects in the 200-million (M) arm (at 3 mo, *p* = .02) but not the 20 or 100M arms. *p Values are for within treatment arm versus baseline. (C) SF-36 Physical Component Score Improvement. The average physical component score improved for subjects in the 100-million (M) (at 1 mo, *p* = .002; at 3 mo, *p* = .03; and at 6 mo, *p* = .03) allo-hMSCs cell-dose group at 1, 3, and 6 mo. *p Values are for within treatment arm versus baseline.

For all cell-dose groups combined, distance walked in 6 minutes increased at 3 months (22.6 m [95% CI: 3.5, 41.7], *p* = .02) and at 6 months (39.3 m [95% CI: 18.7, 60.0], *p* = .001) postinfusion. Subjects in the 100-million allo-hMSC cell-dose group demonstrated the largest improvement from baseline at 3 months (36.6 m [95% CI: 3.5, 69.7], *p* = .03; Figure 2A) and at 6 months (76.6 m [95% CI: 40.8, 112.4], *p* = .0005; Figure 2A). No statistically significant mean increase relative to baseline was seen in the 200-million cell-dose group (Table 4). Assessments of handgrip strength and exercise-induced change in ejection fraction did not reveal significant changes in any of the treatment groups.

**Table 4.** CRATUS Phase 1: Changes From Baseline in Secondary Efficacy Parameters

	Allo-20M (*N* = 5)	Allo-100M (*N* = 5)	Allo-200M (*N* = 5)
6MWD			
3 mo, mean (95% CI)	27.6 m (−5.5, 60.7), *p* = .09	36.6 m (3.5, 69.7), *p* = .03	3.6 m (−29.5, 36.7), *p* = .82
6 mo, mean (95% CI)	37.2 m (1.4, 73.0), *p* = .04	76.6 m (40.8, 112.4), *p* = .0005	4.2 m (−31.6, 40.0), *p* = .80
FEV1			
3 mo, mean (95% CI)	−0.07 L (−0.28, 0.14), *p* = .51	0.17 L (−0.04, 0.38), *p* = .10	0.26 L (0.05, 0.47), *p* = .02
6 mo, mean (95% CI)	−0.01 L (−0.27, 0.25), *p* = .93	0.04 L (−0.22, 0.30), *p* = .76	0.23 L (−0.03, 0.49), *p* = .08
MMSE			
6 mo, median (IQR)	0.5 (3.0), *p* = .75	2.0 (1.0), *p* = .13	0.0 (0.0), *p* = 1.00
TNF-α			
6 mo, mean (95% CI)	−1.2 (−2.7, 0.2), *p* = .09	−3.7 (−5.1, −2.2), *p* = .0001	−3.8 (−5.2, −2.3), *p* = .0001
EQ-5D			
1 mo, median (IQR)	−1.0 (1.0), *p* = .75	−1.0 (2.0), *p* = .25	−1.0 (0.0), *p* = .56
3 mo, median (IQR)	1.0 (3.0), *p* = .69	−1.0 (0.0), *p* = .13	−1.0 (2.0), *p* = .88
6 mo, median (IQR)	1.0 (2.0), *p* = 1.00	−1.0 (1.0), *p* = .13	−1.0 (0.0), *p* = .56
SF-36 Physical Component Score			
1 mo, mean (95% CI)	0.06 (−5.72, 5.84), *p* = .98	10.48 (4.70, 16.26), *p* = .002	0.36 (−5.41, 6.14), *p* = .89
3 mo, mean (95% CI)	−0.87 (−7.01, 5.27), *p* = .76	6.92 (0.78, 13.06), *p* = .03	0.04 (−6.10, 6.18), *p* = .99
6 mo, mean (95% CI)	2.49 (−4.73, 9.70), *p* = .47	7.80 (0.59, 15.02), *p* = .04	−0.10 (−7.32, 7.12), *p* = .98
SF-36 Mental Component Score			
1 mo, mean (95% CI)	3.28 (−3.49, 10.04), *p* = .31	0.83 (−5.93, 7.59), *p* = .79	1.07 (−5.69, 7.84), *p* = .74
3 mo, mean (95% CI)	−5.68 (−13.69, 2.33), *p* = .15	1.13 (−6.88, 9.14), *p* = .76	−0.15 (−8.16, 7.86), *p* = .97
6 mo, mean (95% CI)	−8.07 (−16.79, 0.65), *p* = .07	1.13 (−7.59, 9.84), *p* = .78	5.99 (−2.73, 14.71), *p* = .16

Note: CI = confidence interval; FEV1 = forced expiratory volume in 1 second; IQR = interquartile range; MMSE = Mini-Mental State Examination; 6MWD = 6-minute walk distance; TNF = tumor necrosis factor.

With regards to FEV1, the combined cell-dose groups showed a trend of improvement at 3 months (0.12 L [95% CI: 0.0007, 0.24], *p* = .05) and 6 months (0.09 L [95% CI: −0.06, 0.24], *p* = .23). Subjects in the 200-million allo-hMSC cell-dose group showed the greatest improvement in FEV1 relative to baseline at 3 months postinfusion (0.26 L [95% CI: 0.05, 0.47], *p* = .02) and 6 months postinfusion (0.23 L [95% CI: −0.03, 0.49], *p* = .08; Figure 2B).

Cognitive status, as assessed by the MMSE was evaluated at baseline and 6 months postinfusion. The 100-million cell-dose group exhibited the most improvement in MMSE with a median total score change of 2.0 (interquartile range [IQR]: 1.0–2.0), compared to the 20-million cell-dose group (0.5, IQR: −0.5–2.5) and the 200-million cell-dose group (0.0, IQR: 0.0–0.0), although this was not a significant increase from baseline (*p* = .13). However, there was a statistically significant improvement in the combined cell-dose group from baseline (0.5 [IQR: 0.0–2.0], *p* = .04).

Quality of life was assessed at 1, 3, and 6 months postinfusion by the SF-36 and EQ-5D questionnaires. The SF-36 questionnaire is made up of a physical component score and a mental component score. The 100-million allo-hMSCs cell-dose group showed a significant improvement in the physical component score at 1 month (10.48 [95% CI: 4.70, 16.26], *p* = .002), 3 months (6.92 [95% CI: 0.78, 13.06], *p* = .03), and 6 months (7.80 [95% CI: 0.59, 15.02], *p* = .03; Figure 2C). No significant changes from baseline were found in any of the cell-dose groups for the mental component score. Additionally, there were no significant changes from baseline for the EQ-5D questionnaire.

**Figure 3.**
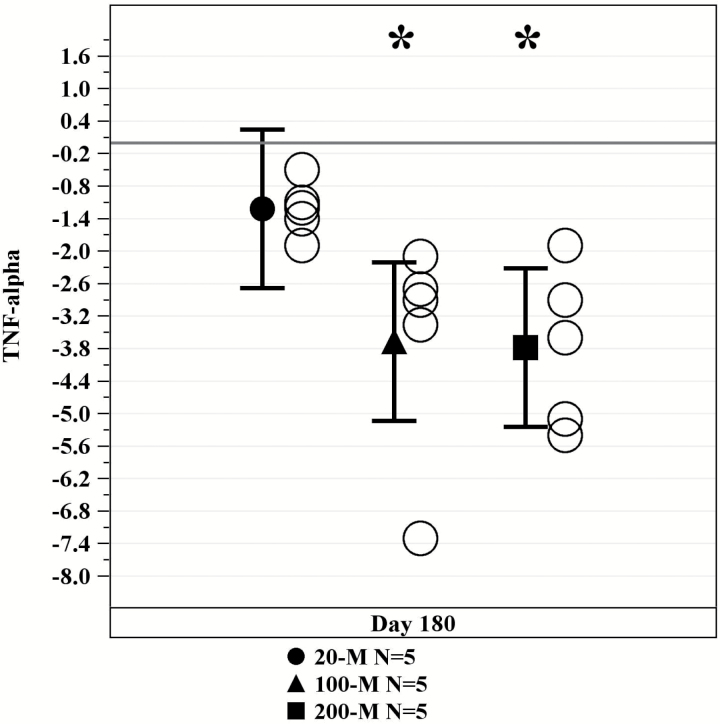
Tumor necrosis factor-α levels decreased with allogeneic mesenchymal stem cell (MSC) treatment. Serum levels significantly decreased over 50% at 6 mo after infusion with allogeneic MSCs in both the 100M and 200M groups (100M and 200M *p* = .0001). *p Values are for within treatment arm versus baseline.

Evaluation of inflammatory marker assessments revealed a significant decrease in TNF-α in the 100-million and 200-million treatment arms (Figure 3). The 20-million allo-hMSC cell-dose group exhibited a moderate change (−1.2 [95% CI: −2.7, 0.2], *p* = .09), whereas the 100-million and 200-million allo-hMSC cell-dose groups showed a more pronounced change from baseline (−3.7 [95% CI: −5.1, −2.2], *p* = .0001) and (−3.8 [95% CI: −5.2, −2.3], *p* = .0001), respectively. No significant changes were seen in CRP, IL-6, fibrinogen, D-dimer, or white blood cell counts in this study.

## Discussion

The major new findings of CRATUS are that intravenous allo-hMSC infusions are safe and well tolerated in elderly individuals with early signs and symptoms of frailty. Importantly, there were improvements in a constellation of parameters that are important predictors of morbidity and mortality in patients with aging frailty.

With no current standard of care for frailty, allo-hMSCs may hold great promise as a cell therapy agent for patients with this syndrome. The underlying basis for positive effects of allo-MSCs are likely due, at least in part, to anti-inflammatory and proregenerative effects (21). In this regard, frailty is characterized by systemic inflammation (17) and low “reserve capacity” of organ systems thought due to diminished endogenous stem cell production (16). Though baseline stem cell levels are difficult to detect, aging in itself is likely associated with reduced stem cells (11,12) and of those which remain viable, there is a loss in their differentiation capacity types (15), which could contribute to a decline in multiorgan physiologic reserve. Replenishment of the body’s stem cell “factory” and/or revitalization of stem cell niches via intravenous infusion of allo-hMSCs may help treat the morbidities associated with aging frailty. Additionally, the decline in function of aged stem cells (12) can be circumvented by administration of young donor allo-hMSCs that do not exhibit senescence or functional impairments that are a characteristic of older donor MSCs (13,16).

The safety of allogeneic MSC therapy has been demonstrated in numerous studies (18–20,22). Additionally, cPRA testing indicated that infusion of allo-hMSCs does not induce a clinically relevant immune response. This may be related to the immunoregulatory properties of MSCs (21), specifically on T-helper and T-regulatory cells (30). Furthermore, our data demonstrate that the allo-hMSCs had an immunomodulatory effect suppressing early and late/chronic T cell activation markers in all treatment groups.

At 6 months postinfusion, we observed a significant decrease in the inflammatory biomarker TNF-α in all cell-dose groups, with the greatest change in the 100- and 200-million groups. These findings corroborate the association between elevated cytokines and systemic inflammation, as well as the role of this marker in the accelerated aging process (24). Indeed, systemic inflammatory biomarkers have been implicated in augmenting cell mitochondrial dysfunction, altered cell-to-cell interactions, and stem cell reserve exhaustion (4). Moreover, elevated inflammatory cytokines like TNF-α have been linked to the disruption of physiological organ systems and their homeostatic properties (24). Other biomarkers, including IL-6 and CRP, did not show any significant decrease following cell therapy in this Phase 1 study, although these cytokines have been previously found to be associated with aging frailty (24,31).

The 6MWD is a validated field test, widely used to assess functional exercise capacity, treatment effectiveness, and prognosis since it is accurate, reproducible, easy to administer, and well-tolerated by patients (32–35). It has been commonly used as a prognostic instrument to assess the functional status of patients with cardiovascular (32,36), pulmonary (33), and more recently, muscular diseases (34). Using the 6MWD to evaluate physical functioning after allo-hMSC intravenous infusion therapy in patients with frailty, we observed significant improvements in distance walked in the 20-million and 100-million cell-dose groups, with the greatest change seen in the 100-million group. Interestingly, we did not find a significant difference in the 200-million cell-dose group. Although safety and tolerability was demonstrated at this cell-dose, significantly valid conclusions can only be made regarding efficacy with additional studies (37). Similarly, in regards to efficacy, it is important to note that there may be an effect in regards to practice with the 6MWD over time. Importantly, the 6MWD test is particularly applicable to frailty as it is an integrated global assessment of patients’ cardiac, respiratory, circulatory, as well as muscular capacities. Age-related skeletal muscle loss, or sarcopenia, can lead to diminished strength, lack of endurance, and low tolerance for physical exertion; all common core clinical presentations of frailty (7). Indeed, the strong association between sarcopenia and functional impairment in older patients has been reported (38). As such, the 6MWD can be used as a meaningful reflection of a frail patient’s ability to perform basic activities of daily living and given the improvement noted in this study, future clinical testing of intravenous allo-hMSC infusion in frail subjects is warranted.

The minimal clinically important difference MCID is defined as the smallest difference in score in the domain of interest, which patients perceive as beneficial and which would mandate a change in patient management (39). In regard to the 6MWD, Perera and colleagues have suggested that an improvement by more than 49 m is considered a “substantial change” (40) and this threshold was indeed exceeded in the 100-million group. However, it is important to consider that although small changes may be statistically significant, they may not be clinically relevant. The MCID is a dynamic and subjective concept, and its derivations are usually estimated for a specific population at a particular stage of recovery (32). Therefore, the fact that 6MWD improves after cell-therapy in this study warrants future clinical testing of intravenous allo-MSC infusion therapy on efficacy outcomes.

Other outcome measurements for efficacy in this Phase 1 trial, including pulmonary function and cognitive function, showed variable results or suggested trends. In regards to the MMSE, the analysis of all cell-dose groups combined showed significant improvement from baseline raising an intriguing possibility that MSCs may improve cognition, a prediction that will require further testing.

Taken together, the dose-finding portion of this study supports the idea that 100-million cells represent the optimal dose level, with no added benefit or loss of effect at the 200-million dose. Interestingly, TNF-α levels appear to be a useful biomarker of efficacy in this population, as they decline in response to all doses of allo-hMSCs. Studies in patients with ischemic cardiomyopathy also suggest a dose threshold effect. However, the small sample size of this Phase 1 and inadequate powering of the secondary outcomes likely contributed to the mixed results noted in regards to pulmonary function and cognition. Phase 2 clinical trials in aging frailty patients with larger sample sizes and blinded, randomized study designs are underway that are designed to confirm or refute the provocative efficacy results shown here. Another limitation of this study was the patient demographics. All subjects were of white race, with no inclusion of African Americans or Hispanics, which would be reasonable to attain in a larger, Phase 2 study sample.

In the United States, the population of individuals 65 years and older is projected to increase to almost 30 times the number recorded in 1990 (3,080,498 persons) (NP2008-T12, U.S. Administration on Aging). Although the overall prevalence of aging frailty has been reported to be near 10%, studies have shown a wide range in the actual prevalence numbers, from less than 5% to 59% (3). This phenomenon is at least in part due to the lack of consensus on the syndrome’s true defining criteria in addition to the array of models used to describe it (41). The various sets of diagnostic models for the syndrome are related to the fact that frailty involves social and psychological components in addition to physical or clinical symptoms (5–7). Therefore, no single model exists that can comprehensively encompass all the essential aspects of the syndrome. As patients with frailty are a major source of medical resource expenditures, early interventions and/or treatments for this disease can improve patient quality of life in addition to lowering healthcare costs (42).

In conclusion, intravenous administration of allo-hMSCs was safe, well tolerated, and free from any concerning adverse events. Furthermore, given the promising results of this study, larger, double-blinded, placebo-controlled clinical trials examining the potential benefits of allo-hMSCs are warranted to further bolster understanding of the efficacy profile of allo-MSCs in individuals with aging frailty.

## Supplementary Material

Supplementary data is available at *The Journals of Gerontology, Series A: Biological Sciences and Medical Sciences* online.

## Funding

This work was supported by the Starr Foundation and the Soffer Family Foundation.

## Conflict of Interest

J.M.H. has a patent for cardiac cell-based therapy; he holds equity in Vestion Inc., maintains a professional relationship with Vestion as a consultant and member of the Board of Directors and Scientific Advisory Board, and is a shareholder in Longeveron LLC. E.A., J.E-K., and A.M. are employees of The Emmes Corporation. A.K. and A.M.L. maintain a professional relationship with Longeveron LLC as consultants. A.A.O., G.G., D.L.D., and A.M. are employees of Longeveron LLC. The other authors declare that they have no competing interests.

## Supplementary Material

Supplementary Material
