# Supplementary material for: Allogeneic Human Mesenchymal Stem Cell Infusions for Aging Frailty
Source: J Gerontol A Biol Sci Med Sci. 2017 Apr 21;72(11):1505–12. doi: 10.1093/gerona/glx056 (PMC5861970; doi:10.1093/gerona/glx056)
Supplement: Supplementary Material [file glx056_suppl_supplementary_material.docx]

**Supplementary Material**

Allo-hMSCs were manufactured at the University of Miami, Interdisciplinary Stem Cell Institute. Donors included males and females between the ages of 20 and 45. Briefly, bone marrow was aspirated from the iliac crest. The mononuclear cell fraction was isolated by Ficoll density gradient separation and washed with Plasma-LyteA containing 1% HAS, and viable cells were seeded in tissue culture flasks containing alpha MEM and 20% FBS. MSCs were isolated based on plastic adherence and expanded for 2 passages. The MSCs were harvest via trypsinization and cryopreserved until date of infusion. A detailed description of donor eligibility, cell harvesting, and culture expansion techniques are published.[^28^](#_ENREF_28) All donors were screened and evaluated by history and physical examination.[^28^](#_ENREF_28) Additionally, informed consent was obtained from all donors and, after hospital discharge, they were contacted by the study team via telephone call for appropriate health status evaluation.

Primary and secondary objectives were to evaluate the safety and efficacy, respectively, of allo-hMSC administration in frail individuals. Safety and tolerability was monitored by adverse events (AEs), serious adverse events (SAEs), and clinical laboratory tests at each visit until 12 months post-infusion. An adverse event was defined as any untoward medical occurrence in a subject temporally associated with the use of a medicinal product, whether or not considered related to the medicinal product (i.e., allo-hMSCs). A serious adverse event was defined as an AE that either resulted in death, was life-threatening (at time of the event), required inpatient hospitalization, prolonged existing hospitalization, resulted in disability and/or incapacity, or resulted in a congenital anomaly/birth defect[^29^](#_ENREF_29).

Immune monitoring was conducted to track any adverse immune reactions to the allo-hMSCs. Calculated panel reactive antibodies (cPRA) against donor human leukocyte antigens (HLA) were measured at baseline and at six-months using Luminex 200. Lymphocytes were stained for T-cells markers of activation CD3, CD69 (early activation) and CD25 (late/chronic activation). All samples were acquired using the LSR-Fortessa-HTS analyzer (BD Pharmigen) and analyzed with the FlowJo V10 software.

Efficacy outcomes were measured at baseline, 3- and 6-months post-infusion. Measurements for efficacy included exercise tolerance and endurance (evaluated via 6-minute walk distance (6MWD) test), diminished handgrip strength (evaluated via dynamometer and short physical performance battery (SPPB) assessment), health outcome quality of life (evaluated via the Euro-QOL 5 Dimensions (EQ-5D) questionnaire), and patient-reported health status (evaluated via the 36-Item Short Form (SF-36) survey). The following additional assessments were also used for efficacy outcome measurements: Mini-Mental State Examination (MMSE) score, forced expiratory volume in 1 second (FEV1), death from any cause, exercise change in ejection fraction (measured with dobutamine stress test), and a panel of inflammatory markers, including C-reactive protein (CRP), interleukin-6 (IL-6), tumor necrosis factor (TNF)-α, fibrinogen, D-dimer, and white blood cell count.

**Statistical Methods**

Continuous variables are summarized using means and standard deviations, or medians and interquartile range, as appropriate. Frequency and percentages (based on the non-missing sample size) of observed levels are reported for categorical measures. For outcomes that were collected at multiple follow-up visits, a mixed model for repeated measures was used with treatment group, time, and a group-by-time interaction. As this was not a randomized study, analyses focus on within-group effects described using model-estimated contrasts. Outcomes that were highly skewed were analyzed using a Wilcoxon signed rank test for within-group effects. Outcomes for T cell activation markers were analyzed using a two-way Anova and Sidak’s multiple comparisons test. All statistical tests were performed at α=0.05 using two-sided tests. P-values are reported to two significant digits. No adjustments for multiple comparisons were made due to the early phase, exploratory nature of the study.[^29^](#_ENREF_29) Results from these exploratory analyses provide preliminary information on relationships in the data that could be subject to more rigorous future studies.

Supplementary Table 1. CRATUS Phase I: 12-Month AE Summary

|  | | **Dose Level** | | | | | |  | |
| --- | --- | --- | --- | --- | --- | --- | --- | --- | --- |
|  | | **Allo-20M (N=5)** | | **Allo-100M (N=5)** | | **Allo-200M (N=5)** | | **Total (N=15)** | |
|  | | **Events** | **Patients** | **Events** | **Patients** | **Events** | **Patients** | **Events** | **Patients** |
| **System Organ Class** | **MedDRA Preferred Term** | **n (%)** | **n (%)** | **n (%)** | **n (%)** | **n (%)** | **n (%)** | **n (%)** | **n (%)** |
| Ear and labyrinth disorders | Inner ear inflammation | 1 (7.1%) | 1 (20.0%) |  |  |  |  | 1 (3.8%) | 1 (6.7%) |
| Gastrointestinal disorders | Diarrhoea |  |  | 1 (16.7%) | 1 (20.0%) |  |  | 1 (3.8%) | 1 (6.7%) |
| General disorders and administration site conditions | Infusion site extravasation | 1 (7.1%) | 1 (20.0%) |  |  |  |  | 1 (3.8%) | 1 (6.7%) |
|  | Sudden cardiac death |  |  |  |  | 1 (16.7%) | 1 (20.0%) | 1 (3.8%) | 1 (6.7%) |
| Immune system disorders | Drug hypersensitivity | 1 (7.1%) | 1 (20.0%) |  |  |  |  | 1 (3.8%) | 1 (6.7%) |
| Infections and infestations | Cellulitis | 1 (7.1%) | 1 (20.0%) |  |  |  |  | 1 (3.8%) | 1 (6.7%) |
|  | Influenza |  |  | 1 (16.7%) | 1 (20.0%) |  |  | 1 (3.8%) | 1 (6.7%) |
|  | Oral herpes |  |  |  |  | 1 (16.7%) | 1 (20.0%) | 1 (3.8%) | 1 (6.7%) |
|  | Upper respiratory tract infection | 1 (7.1%) | 1 (20.0%) | 1 (16.7%) | 1 (20.0%) | 1 (16.7%) | 1 (20.0%) | 3 (11.5%) | 3 (20.0%) |
|  | Urinary tract infection | 1 (7.1%) | 1 (20.0%) |  |  |  |  | 1 (3.8%) | 1 (6.7%) |
| Injury, poisoning and procedural complications | Fall | 1 (7.1%) | 1 (20.0%) |  |  |  |  | 1 (3.8%) | 1 (6.7%) |
|  | Incisional hernia | 1 (7.1%) | 1 (20.0%) |  |  |  |  | 1 (3.8%) | 1 (6.7%) |
|  | Wrist fracture |  |  | 1 (16.7%) | 1 (20.0%) |  |  | 1 (3.8%) | 1 (6.7%) |
| Metabolism and nutrition disorders | Hypercalcaemia | 1 (7.1%) | 1 (20.0%) |  |  |  |  | 1 (3.8%) | 1 (6.7%) |
| Musculoskeletal and connective tissue disorders | Bursitis |  |  | 1 (16.7%) | 1 (20.0%) |  |  | 1 (3.8%) | 1 (6.7%) |
| Neoplasms benign, malignant and unspecified (incl cysts and polyps) | Basal cell carcinoma | 1 (7.1%) | 1 (20.0%) |  |  |  |  | 1 (3.8%) | 1 (6.7%) |
| Nervous system disorders | Neuropathy peripheral |  |  |  |  | 1 (16.7%) | 1 (20.0%) | 1 (3.8%) | 1 (6.7%) |
|  | Piriformis syndrome | 1 (7.1%) | 1 (20.0%) |  |  |  |  | 1 (3.8%) | 1 (6.7%) |
| Respiratory, thoracic and mediastinal disorders | Dyspnoea | 1 (7.1%) | 1 (20.0%) |  |  |  |  | 1 (3.8%) | 1 (6.7%) |
|  | Oropharyngeal pain | 1 (7.1%) | 1 (20.0%) |  |  |  |  | 1 (3.8%) | 1 (6.7%) |
| Skin and subcutaneous tissue disorders | Ecchymosis |  |  |  |  | 1 (16.7%) | 1 (20.0%) | 1 (3.8%) | 1 (6.7%) |
|  | Skin lesion |  |  |  |  | 1 (16.7%) | 1 (20.0%) | 1 (3.8%) | 1 (6.7%) |
| Surgical and medical procedures | Carpal tunnel decompression |  |  | 1 (16.7%) | 1 (20.0%) |  |  | 1 (3.8%) | 1 (6.7%) |
| Vascular disorders | Hypertension | 1 (7.1%) | 1 (20.0%) |  |  |  |  | 1 (3.8%) | 1 (6.7%) |
| Total |  | 14 (100.0%) | 5 (100.0%) | 6 (100.0%) | 4 (80.0%) | 6 (100.0%) | 3 (60.0%) | 26 (100.0%) | 12 (80.0%) |
